# Causal Connectivity Maps Derived from Single-Pulse Interleaved TMS/fMRI

**DOI:** 10.21203/rs.3.rs-7158945/v1

**Published:** 2025-08-11

**Authors:** Lison Bossus, Jess Dickson, Camille Blaine, Hasti Khalilkhani, Ahsan Khan, Desmond J. Oathes

**Affiliations:** University of Pennsylvania; University of Pennsylvania; University of Pennsylvania; University of Pennsylvania; University of Pennsylvania; University of Pennsylvania

## Abstract

Here we employed interleaved single-pulse transcranial magnetic stimulation with functional MRI (spTMS/fMRI) to generate causal activation maps targeting the left hemisphere according to two sets of personalized resting connectivity targeting approaches in over 80 participants. Voxelwise event-related fMRI BOLD maps quantified TMS-evoked brain responses to each stimulation site. Group-level maps controlled for head motion, scalp discomfort, and somatosensory effects, ensuring specificity of the intended brain responses to stimulation. Stimulation of frontal regions targeting the subgenual anterior cingulate cortex (sgACC) induced BOLD responses in the sgACC and modulated distributed cortical and subcortical regions. Similarly, ventrolateral targets led to an average negative BOLD response in the amygdala target and numerous other distributed brain regions. ROI-based analyses revealed no significant group differences (depressed vs. healthy control) in evoked responses within the sgACC or amygdala. These results validate that image-guided TMS can causally engage distributed brain regions, supporting its utility for circuit and network-based neuromodulation. The publicly available causal connectivity maps might be used to guide future research linking cortical circuit maps with fMRI and/or behavioral outcomes.

## Introduction

- fMRI is correlational and limited in causal inference

Standard functional MRI has been used to identify co-activation/temporal correlation between regions, suggesting potential connectivity, but it does not easily demonstrate directionality in which region influences another. Advances that combine large normative human fMRI connectivity (correlation) maps with data from lesions in human patients has shown promise as a tool to understand how distributed brain damage might result in similar neuropsychiatric symptom manifestations (Joutsa et al., 2022). One limitation of this approach is that applying these maps at the individual patient level for potential treatment targeting is not yet established. By contrast, combining perturbation methods with neuroimaging at the individual patient level has the potential to uncover directional relationships that build on traditional correlational approaches (Oathes, Balderston, et al., 2021; Roalf et al., 2024).

- Structural techniques and some limits

Diffusion tensor imaging (DTI) and diffusion spectrum imaging (DSI) are commonly used to map structural brain connections in neuropsychiatric research as well as clinical applications such as deep brain stimulation targeting (Granziera et al., 2021; Nagaraja, 2021; Riva-Posse et al., 2018; Roalf et al., 2024) While it provides valuable anatomical insights, diffusion may fail to capture indirect yet functionally meaningful connections. There is substantial evidence suggesting that these regions interact through polysynaptic pathways involving multiple neural relays. Because diffusion MRI is most accurately employed for resolving direct structural tracts, it can easily overlook anatomically complex circuits (Al-Sharif et al., 2025).

- Image-guided Transcranial Magnetic Stimulation

Functional connectivity MRI (fcMRI) is increasingly used to guide TMS targeting, with the goal of reaching distributed regions such as the subgenual anterior cingulate cortex (sgACC), amygdala or anterior insula cortex indirectly via correlated cortical partners (Cole et al., 2020, 2022; Duprat et al., 2025; Morriss et al., 2024; Oathes et al., 2023; Oathes, Zimmerman, et al., 2021; Rooij et al., 2024; Soleimani et al., 2025; Sydnor et al., 2022). However, aspiring to engage a remote brain target with TMS does not prove that the target was effectively engaged or that the targeted circuit was modulated even in the context of highly effective image-guided rTMS treatment. In fact, the benefit of treatment may be better represented by brain changes in regions not targeted with fMRI-guided TMS treatment (Batail et al., 2023). Without validation, it is unclear whether stimulation actually engages or modulates the desired target.

Interleaved or concurrent TMS/fMRI addresses this limitation by enabling causal testing of functional circuit models. It allows researchers to determine whether a given cortical site influences specific downstream regions and to observe interactions within and between large-scale brain networks. This noninvasive and in vivo evidence of circuit engagement confirms whether or not stimulation reaches the intended functional region or network.

In addition to improving our understanding of directional and causal brain connectivity, this approach offers a practical advantage for TMS interventions: using the same stimulation tool to both validate functionally relevant targets and to modulate them enhances a mechanistic understanding of neuropsychiatric disorders and especially how to treat them effectively. Biomarkers alone might uncover compensatory processes that are not ideal targets for treatment. In addition, a putative biomarker may represent a risk-factor for an illness that may be immutable by any known intervention. The same intervention applied using the same fMRI-guided targeting approach might influence more than one symptom cluster (e.g. depressed mood vs. anxious arousal) but through modulation of different downstream connected brain regions (Oathes et al., 2023). Ultimately, adding imaging recordings to brain stimulation increases the likelihood of effectively modulating symptom-relevant networks and achieving better clinical outcomes (Oathes, Balderston, et al., 2021; Roalf et al., 2024).

### Prior applications of TMS/fMRI as a causal mapping tool

- Combined imaging with stimulation allows mapping of causal pathways

Single pulse stimulation protocols allow for the assessment of the brain’s immediate response to focal stimulation: when a single TMS pulse is applied to a specific site, it briefly perturbs neural activity in that area. By measuring the resulting BOLD responses in the brain using fMRI, we can identify which regions show a time-locked response. These evoked responses reflect functionally connected networks that are engaged by the stimulation.

Because the timing and location of the pulse are known, any observed brain activity in response to the pulse can be interpreted as a direct consequence of that stimulation. We can then draw causal inferences about which brain areas are influenced downstream by the target site.

- Single-pulse and repetitive TMS/fMRI protocols offer complementary strengths

With single pulse TMS, although the stimulation is reasonably focal, direct activation under the TMS coil is highly circumscribed – as shown by Romero et al. (2019) using electrophysiology, who reported that only a small number of neurons are depolarized under the coil. As a result, single pulse TMS produces limited neuronal activation, making it difficult to detect local BOLD signal under the TMS coil with fMRI (Bergmann et al., 2021; Rafiei et al., 2021).

Evidence suggests that this absence of detectable signal to single TMS pulses does not imply a lack of neural effect. Functionally, single TMS pulses can reliably evoke strong consistent muscle contractions in specific distal muscles when stimulating corresponding regions of the primary motor cortex (M1). Rafiei et al. (2021) demonstrated that, despite the lack of local BOLD increase, single-pulse TMS elicits distributed multivoxel fMRI BOLD responses. Similarly, we found that single pulse (sp) TMS/fMRI can effectively engage distributed canonical resting brain networks as well as demonstrate causal antagonism between putative task-positive and default mode networks (Chen et al., 2013).

In addition, repetitive TMS (rTMS) bursts can also be interleaved with fMRI recordings, including with interslice pairings that allow acquisition of fMRI in the context of clinically used rTMS patterns without corrupting the imaging data. Hardware configurations, scan protocols, and timing optimization for these challenging protocols are being explored (Jackson et al., 2025; Tik et al., 2021). Thus far, the higher frequency rTMS protocols with surface coils mounted to the TMS coil have been most consistent in demonstrating fMRI BOLD directly under the TMS coil (Navarro de Lara et al., 2015; Navarro De Lara et al., 2017; Tik et al., 2017; Xia et al., 2024). One interpretive challenge for rTMS/fMRI is the extent to which the average BOLD response over a run reflects neuromodulation as opposed to simple circuit engagement given that rTMS is designed to change brain activity.

- Clinical relevance of TMS/fMRI

Causal connectivity maps derived from TMS/fMRI have been associated with clinical response to rTMS interventions in depression. In our work, we have shown that spTMS/fMRI (circuit engagement) evoked responses in the sgACC were a positive prognostic predictor of depression improvement to rTMS of the same fMRI-guided target (Oathes et al., 2023). Another group similarly found that rTMS/fMRI at 1Hz induced a network-level connectivity change that predicted 30% of the variance in depression improvement to 1Hz rTMS treatment (Ge et al., 2022). In our prior studies, we found also that the change in sgACC evoked response from pre to post-intervention (circuit modulation) was associated with depression improvement, showed target specificity (compared with an amygdala target), demonstrated symptom specificity of depression improvement associated with sgACC evoked response change, and showed that sgACC positive FC targets evoked stronger sgACC responses in patients compared with anticorrelated targets (Duprat et al., 2025; Oathes et al., 2023).

### Scientific and clinical promise

- Toward causal methods

Tools such as optogenetics/fMRI (Ferenczi et al., 2016), cortico-cortico evoked potentials (Seguin et al., 2023), deep brain stimulation/fMRI (Boutet et al., 2021), and TMS/stereo EEG (Trapp et al., 2024) can offer complementary evidence of circuit engagement and modulation in clinically relevant networks. The advantage of TMS/fMRI (and TMS/EEG) is that the methods are non-invasive and so could be reasonably applied to nearly any neuropsychiatric patient and healthy individual included in a research study. TMS/EEG may be most advantageous to study signals that require the greatest temporal precision (Parmigiani et al., 2025) whereas TMS/fMRI might best capture differential regional and brain network responses.

### Framing

In our prior work, we used specific subcortical regions of interest (sgACC and amygdala) to seed functional connectivity (resting correlation) maps at the cortical surface personalized for each participant. Given these *a priori* defined intended downstream targets, we initially validated image-guided TMS coil placement by measuring sgACC and amygdala evoked responses according to interleaved spTMS/fMRI. Here we present spTMS/fMRI maps combined across several of our published studies (Duprat et al., 2025; Oathes et al., 2023; Sydnor et al., 2022) in order to define at the whole-brain level TMS-evoked fMRI responses with TMS coil placement spanning either left frontal regions or left ventrolateral regions that were defined by sgACC and amygdala (basolateral) connectivity, respectively.

In order to demonstrate robust TMS/fMRI maps, we threshold the images by a minimum number of individual participants who had TMS-evoked % signal change of a minimum intensity for that voxel. We further masked the maps by a minimum effect size, residualize the data for confounds (somatosensory evoked response and discomfort ratings), then present final maps in t-statistic units. We further point out the sgACC and amygdala evoked responses that survive these thresholds as well as compare the healthy and depressed patient evoked responses in these regions of interest.

In terms of use cases for these spTMS/fMRI causal connectivity atlases, we anticipate that some fMRI researchers may want to confine their whole brain analyses by limiting the search space to regions connected downstream, causally, from these broad cortical locations. Similarly, TMS researchers with fMRI data may want to look for clinical associations within regions known here to be effectively engaged with TMS. The sgACC and amygdala regions of interest that show robust engagement to TMS may also be used for refined seed-based connectivity in TMS studies or in studies like ours optimizing spTMS/fMRI approaches in regions like the sgACC known to have lower signal-to-noise ratios compared with other brain areas.

## Methods

### Participants and Datasets

We included participants from multiple prior TMS/fMRI studies conducted at the University of Pennsylvania, each approved by the Institutional Review Board. All participants provided informed consent consistent with the Declaration of Helsinki. Participants were between 18 and 56 years old and were either healthy controls (HC) or diagnosed with major depressive disorder (MDD). Individuals with neurological or psychiatric conditions (for healthy controls), or those using psychoactive substances, were excluded.

For each study, participants underwent a baseline structural and functional MRI session, followed by one or more single-pulse TMS/fMRI sessions targeting specific brain regions such as the subgenual anterior cingulate cortex (sgACC) and the basolateral amygdala (BLA). If participants had completed more than one TMS/fMRI session, only the first session was retained to ensure consistent sampling.

In total, activation maps from 105 participants (77 HC and 28 MDD) were included in the sgACC analysis, and 88 participants (58 HC and 30 MDD) in the BLA analysis.

### MRI baseline

All MRI data were collected using a 3T Siemens Prisma scanner. Baseline anatomical and resting-state scans were acquired with a 64-channel head coil. Each participant underwent two multiband resting-state fMRI runs with reverse phase encoding directions (anterior-to-posterior and posterior-to-anterior). Acquisition parameters were as follows: TR = 800 ms, TE = 37 ms, flip angle = 52°, field of view = 208 mm, voxel size = 2×2×2 mm, 72 interleaved axial slices (no gap), 420 volumes per run. Participants were instructed to maintain visual fixation on a central cross while remaining still with their eyes open.

Structural data included a T1-weighted MPRAGE image (TR = 2400 ms, TI = 1060 ms, TE = 2.24 ms, flip angle = 8°, voxel size = 0.8 mm isotropic, FOV = 256 mm, GRAPPA acceleration, 208 slices).

Baseline functional connectivity values used for targeting were computed from seed regions corresponding to the subgenual anterior cingulate cortex (sgACC) and the basolateral amygdala (BLA). These seeds were transformed into each participant’s native T1 space to identify individualized stimulation targets ipsilateral to TMS application.

### TMS Target Sites

**Figure 1:**
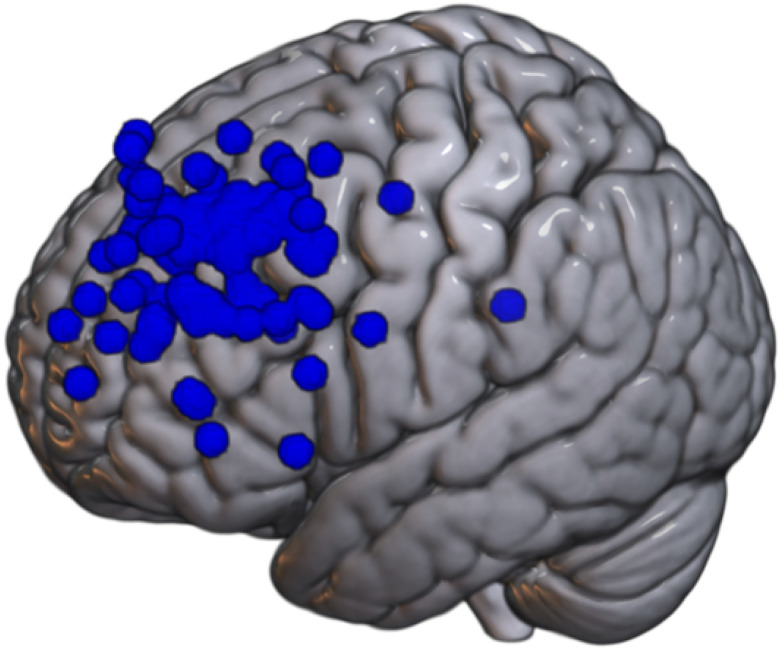
Cortical targets for sgACC

**Figure 2:**
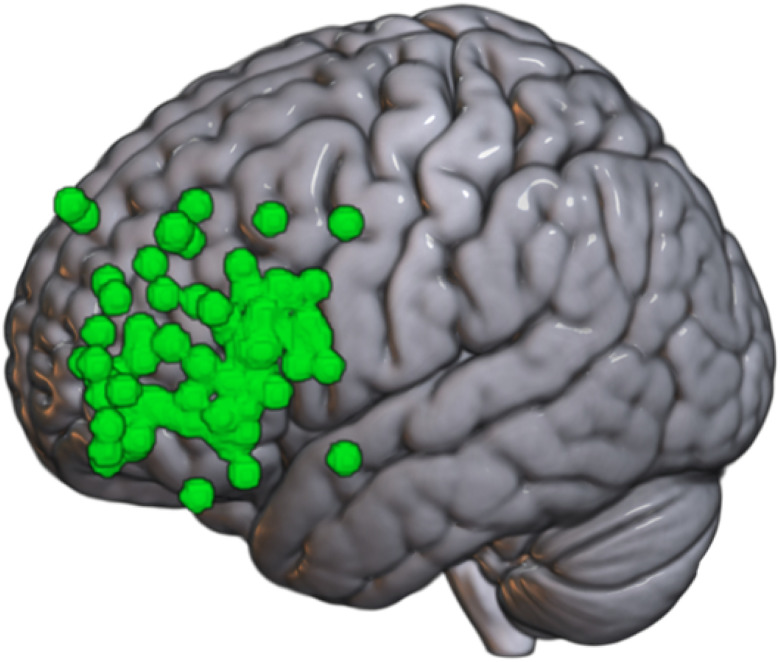
Cortical targets for BLA

Target sites varied across datasets but were primarily located in frontal regions functionally connected to the subgenual anterior cingulate cortex (sgACC) and ventrolateral sites for the basolateral amygdala (BLA). Seed regions for the sgACC and BLA were individually transformed into native space to identify individualized stimulation targets ipsilateral to TMS application. All coordinates were later transformed to MNI space to allow for cataloging the individual cortical TMS targets (see Fig. 1–2).

For TMS/fMRI, neuronavigation was first used to accurately identify each participant’s stimulation target based on their baseline connectivity. These target locations were then marked on a swim cap worn by the participant during MRI scanning.

### Interleaved TMS/fMRI

For TMS within the MRI, a series of single pulses per run were delivered using an MRI-compatible Magventure MRI-B91 air-cooled TMS coil connected to a Magpro X100 stimulator (Magventure Farum, Denmark).

Resting motor threshold (rMT) was determined inside the MRI room. It was defined as the minimal stimulation intensity required to evoke a visible movement in the right hand (FDI or APB) in at least 5 out of 10 stimulations. Stimulation intensity for the TMS/fMRI sessions was then set to 120% of this motor threshold to ensure sufficient cortical engagement during single-pulse stimulation.

TMS/fMRI runs used an echo-planar imaging (EPI) sequence optimized for single-pulse TMS, with the following parameters: TR = 2000 ms, TE = 30 ms, flip angle = 75°, field of view = 192 mm, voxel size = 3×3×4 mm^3^, 32 interleaved axial slices, and 178 volumes. Each TMS/fMRI run included 12 mini-blocks and 71 total pulses, delivered in 400-ms gaps interleaved with fMRI volume acquisitions to allow for hemodynamic response resolution and to avoid interference with image acquisition.

TMS pulses and MRI volumes each individually were triggered via transistor-transistor logic (TTL) signals sent through a parallel port by E-prime 2.0 software (Psychology Software Tools, Sharpsburg, Pennsylvania, USA).

### MRI processing

All MRI data were preprocessed using the eXtensible Connectivity Pipeline (XCP Engine) designed to run FMRI Expert Analysis Tool (FEAT; version 6.0.0). Functional volumes were corrected for head motion using FSL’s MCFLIRT and spatially smoothed with a 5-mm full-width at half-maximum (FWHM) Gaussian kernel. Boundary-based registration was used to align functional data to each participant’s anatomical T1-weighted image, and images were then normalized to MNI space using nonlinear transformations computed with Advanced Normalization Tools (ANTs).

Structural T1-weighted images were segmented using ANTs cortical thickness pipeline.

For baseline resting-state fMRI data, the first two volumes were discarded to allow for scanner equilibration. Functional images were then realigned to the volume with the least motion. Skull stripping was performed using FSL’s BET, and AFNI’s 3dDespike was applied to detect and interpolate outlier time points. The data were demeaned, linearly and polynomially detrended, and band-pass filtered between 0.01 and 0.08 Hz. Confound regression was performed using a 36-parameter model including motion estimates, white matter, cerebrospinal fluid, and global signal (Ciric et al., 2017).

For TMS/fMRI data, a high-pass temporal filter (cutoff = 100 s) was applied and the preprocessing pipeline followed the same general steps described above, including anatomical registration, and normalization to MNI space.

### ROI-Based Analysis

Region-of-interest (ROI) analyses focused on the sgACC and BLA. For each participant, average TMS-evoked signal change was extracted from individually defined or atlas-based ROIs. We then tested whether the mean activation within these regions differed as a function of diagnostic group (MDD vs. healthy controls) using between-group statistical comparisons, conducted in RStudio (v4.5.0).

### Software and Statistical Analyses of activation maps

For each participant, we generated a voxelwise activation map quantifying the neural response to TMS stimulation, expressed as percent signal change relative to the implicit baseline.

To characterize stimulation-induced activation patterns at the group level, individual activation maps were concatenated into a single four-dimensional image. A general linear model (GLM) was constructed to estimate the effect of stimulation while accounting for potential confounding factors. The model included the following covariates of no interest: average head motion (framewise displacement), mean discomfort ratings reported after the scan, and mean somatosensory activation (average across entire somatosensory mask from the Yeo 7-network atlas). These covariates were included to ensure that the resulting activation patterns reflected neural responses specifically attributable to transcranial magnetic stimulation (TMS), rather than to discomfort or other nonspecific sensory effects from TMS.

A voxel was considered reliably activated if at least 75% of participants showed a TMS-evoked percent signal change of at least 0.2% at that location. Additionally, a minimum group-level effect size of 4.0 was required to include a voxel in the final activation mask. For the sgACC ROI, a separate mask was taken by taking the overlap between the whole-brain image and the subcallosal cingulate (25%+ probability) from the Harvard-Oxford atlas and with z-dimension coordinates truncated below − 12.5. The overlap between the basolateral seed mask (derived from the Juelich atlas) was similarly used to define the ‘amygdala’ evoked response region of interest from the whole-brain result. Both are separately uploaded to neurovault in addition to the whole brain atlases.

## Results

TMS was delivered over individualized cortical targets selected via TMS/fMRI to engage either the subgenual anterior cingulate cortex (sgACC) or the basolateral amygdala (BLA). Prefrontal targets were chosen based on their functional connectivity to the sgACC, while ventromedial targets were selected to modulate the BLA.

**Figure 3:**
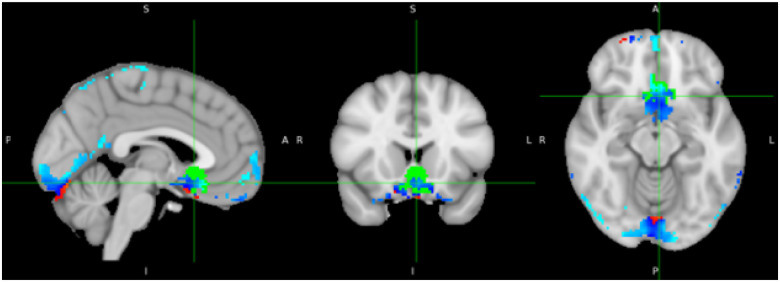
Whole-brain residualized T-statistic map showing regions significantly modulated following transcranial magnetic stimulation (TMS) applied to prefrontal sites targeting the subgenual anterior cingulate cortex (sgACC). Maps are residualized for head motion, somatosensory ratings, and pain perception to control for potential confounding factors. Warm colors represent increased BOLD signal, and cool colors indicate decreased BOLD signal. The sgACC target ROI is overlaid in green for reference.

Stimulation of sgACC FC sites resulted in a negative fMRI BOLD response of the sgACC (Fig. 3), indicating successful engagement of the target circuit. Beyond the sgACC, stimulation led to additional BOLD responses in the frontal medial cortex, the occipital lobe (particularly the lingual gyrus) as well as in the inferior temporal gyrus, the fornix, and other areas, pointing to a broader modulation of downstream networks.

**Figure 4:**
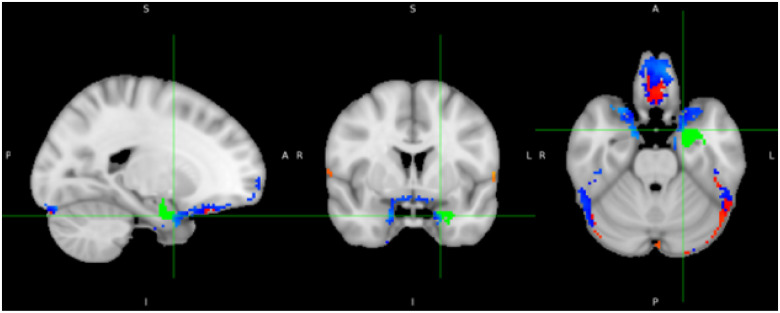
Whole-brain residualized T-statistic map showing regions significantly modulated following transcranial magnetic stimulation (TMS) applied to ventromedial sites targeting the basolateral amygdala. Maps are residualized for head motion, somatosensory ratings, and pain perception to control for potential confounding factors. Warm colors represent increased BOLD signal, and cool colors indicate decreased BOLD signal). The BLA target ROI is overlaid in green for reference.

Similarly, stimulation of ventrolateral sites lead to a negative BOLD response in the amygdala (Fig. 4), consistent with effective engagement of the amygdala through cortical entry points. Additional activity changes were detected in the frontal medial cortex, the occipital lobe (lingual gyrus) as well as in the inferior temporal gyrus, the fornix, and the secondary somatosensory cortex, potentially reflecting downstream effects on distributed circuits.

To investigate whether average evoked fMRI responses in the targeted regions differed between healthy controls (HC) and individuals with major depressive disorder (MDD), we compared the sgACC and BLA ROIs between the two groups. No significant difference was observed in sgACC fMRI BOLD between HC and MDD participants (*t*(55.88) = 0.63, *p* = 0.53, 95% CI = [−0.12, 0.23]; HC mean = − 0.22, MDD mean = − 0.28). Similarly, BLA response did not significantly differ between groups (*t*(58.13) = 1.42, *p* = 0.16, 95% CI = [−0.04, 0.24]; HC mean = − 0.16, MDD mean = − 0.25).

## Discussion

Given the correlational nature of neuroimaging that does not incorporate perturbation tools as well as the shortcoming of most TMS studies to understand at the individual participant/patient level what effects TMS has on the brain, there is a clear justification to complement measures of brain “connectivity” with tools such as interleaved or concurrent TMS/fMRI. Here we offer a robust (thresholded) and reliable (consistent across individuals) set of causal connectivity maps generated using interleaved single-pulse TMS/fMRI across more than 100 participants. One atlas was generated with TMS coil positions personalized based on seed-based fMRI connectivity between the subgenual anterior cingulate cortex and the cortical surface where TMS was applied. The second map was generated similarly but seeding a subregion of the amygdala (basolateral). The sgACC targeting tended to be more medial/dorsal compared with the amygdala targets that tended to be more ventrolateral. For both sets of analyses, we found that the intended subcortical target was engaged consistent with our prior work (Duprat et al., 2025; Oathes et al., 2023; Oathes, Zimmerman, et al., 2021; Sydnor et al., 2022). The distributed causal connectivity maps here presented builds on these *a priori* but more limited analyses.

Resting fMRI connectivity was used to target TMS coil positions which we acknowledge creates a dependence on a measure that may not be ideal for demonstrating ‘causal’ relationships between nodes of a network or ends of a connected circuit. Improvements in resting fMRI acquisition and processing are still under development and may yield superior prediction of TMS induced neuromodulation and symptom change. In our work, we found that only resting connectivity acquired at a timepoint close to the implementation of the rTMS intervention predicted depression improvement in patients (Oathes et al., 2023). TMS/fMRI, however, was a superior predictor of depression improvement. When resting FC (sgACC to site of stimulation) was entered into the same predictive model as TMS-evoked sgACC fMRI BOLD response, FC was no longer a significant predictor whereas TMS/fMRI maintained its predictive accuracy and statistical significance.

spTMS/fMRI applied to a single node of a network is effective at engaging the distributed brain representations of that network (Chen et al., 2013). Nevertheless, a limitation of the technique is that TMS can only be effectively applied at the cortical surface and so other methods for directly modulating deep brain regions (deep brain stimulation, focused ultrasound) may complement neuromodulation tools like TMS that rely on having a surface accessible stimulation target.

It is not the case that personalized image-guided TMS will always yield stronger behavioral or clinical effects compared with less personalized scalp-based targets (Kaster et al., 2023; Morriss et al., 2024). To understand when and how image-guided TMS is effective, concurrent TMS/fMRI can be an important source of evidence (Ge et al., 2022; Oathes et al., 2023). It is also not the case that clinically effective image-guided TMS proves that a specific intended circuit was modulated (Batail et al., 2023; Gajawelli et al., 2024). Again, TMS/fMRI can be used to validate brain circuit target engagement as well as modulation (Oathes et al., 2023). Various groups, including our own, have unique image processing pipelines to generate personalized targeting to presumably access the same brain target as in the quest for TMS cortical sites thought to optimally target the sgACC (Cash et al., 2019; Cole et al., 2020; Duprat et al., 2025; Fox et al., 2012; Kong et al., 2022; Luber et al., 2017; Oathes et al., 2023; Oathes, Zimmerman, et al., 2021). It would be a powerful validation step to test each pipeline’s targeting by comparing within-subjects sgACC evoked responses with TMS/fMRI before the trouble and expense of conducting a full clinical trial with imaging outcome data. This approach is supported by our TMS/fMRI prognostic prediction evidence (Oathes et al., 2023). Expanding on this theme, various “connectivity” brain atlases might similarly be compared and contrasted using causal TMS/fMRI mapping evidence.

In summary, we here present the largest causal connectivity atlas published to date using whole brain fMRI images in response to single TMS pulse mapping. We hope that this database and especially the approach from which it derives might inspire more cognitive and clinical neuroscientists to implement these tools. For additional considerations of research questions addressable (Oathes, Balderston, et al., 2021; Roalf et al., 2024), limitations and confounds, as well as hardware and imaging optimization (Lueckel et al., 2023), we encourage readers to consult a recently published TMS/fMRI consensus paper generated by an international group spanning basic and clinical research applications (Woolgar et al., 2025).

## Data Availability

The data that support the findings of this study are available from the corresponding author, upon reasonable request with an approved data use agreement.

## References

[1] Al-Sharif N. B., Zavaliangos-Petropulu A. & Narr K. L. A review of diffusion MRI in mood disorders: Mechanisms and predictors of treatment response. Neuropsychopharmacology 50 (1), 211–229. 10.1038/s41386-024-01894-3 (2025). PMC11525636 38902355

[2] Batail J. M. Network effects of Stanford Neuromodulation Therapy (SNT) in treatment-resistant major depressive disorder: A randomized, controlled trial. Translational Psychiatry. 13, 240. 10.1038/s41398-023-02537-9 (2023). 37400432 PMC10318050

[3] Bergmann T. O. Concurrent TMS-fMRI for causal network perturbation and proof of target engagement. NeuroImage 237, 118093. 10.1016/j.neuroimage.2021.118093 (2021). 33940146

[4] Boutet A. Predicting optimal deep brain stimulation parameters for Parkinson’s disease using functional MRI and machine learning. Nat. Commun. 12 (1), 3043. 10.1038/s41467-021-23311-9 (2021). 34031407 PMC8144408

[5] Cash R. F. H. Subgenual Functional Connectivity Predicts Antidepressant Treatment Response to Transcranial Magnetic Stimulation: Independent Validation and Evaluation of Personalization. Biol. Psychiatry. 86 (2), e5–e7. 10.1016/j.biopsych.2018.12.002 (2019). 30670304

[6] Chen A. C. Causal interactions between fronto-parietal central executive and default-mode networks in humans. Proceedings of the National Academy of Sciences, 110(49), 19944–19949. (2013). 10.1073/pnas.1311772110 PMC3856839 24248372

[7] Ciric R. Benchmarking of participant-level confound regression strategies for the control of motion artifact in studies of functional connectivity. NeuroImage 154, 174–187. 10.1016/j.neuroimage.2017.03.020 (2017). 28302591 PMC5483393

[8] Cole E. J. Stanford Neuromodulation Therapy (SNT): A Double-Blind Randomized Controlled Trial. Am. J. Psychiatry. 179 (2), 132–141. 10.1176/appi.ajp.2021.20101429 (2022). 34711062

[9] Cole E. J. Stanford Accelerated Intelligent Neuromodulation Therapy for Treatment-Resistant Depression. Am. J. Psychiatry. 177 (8), 716–726. 10.1176/appi.ajp.2019.19070720 (2020). 32252538

[10] Duprat R. J. Resting fMRI-guided TMS evokes subgenual anterior cingulate response in depression. NeuroImage 305, 120963. 10.1016/j.neuroimage.2024.120963 (2025). 39638081 PMC11887861

[11] Ferenczi E. A. Prefrontal cortical regulation of brainwide circuit dynamics and reward-related behavior. Sci. (New York N Y). 351 (6268), aac9698. 10.1126/science.aac9698 (2016). PMC4772156 26722001

[12] Fox M. D., Buckner R. L., White M. P., Greicius M. D. & Pascual-Leone A. Efficacy of transcranial magnetic stimulation targets for depression is related to intrinsic functional connectivity with the subgenual cingulate. Biol. Psychiatry. 72 (7), 595–603. 10.1016/j.biopsych.2012.04.028 (2012). 22658708 PMC4120275

[13] Gajawelli N. Increased anti-correlation between the left dorsolateral prefrontal cortex and the default mode network following Stanford Neuromodulation Therapy (SNT): Analysis of a double-blinded, randomized, sham-controlled trial. Npj Mental Health Res. 3 (1), 35. 10.1038/s44184-024-00073-y (2024). PMC11227523 38971869

[14] Ge R. Predictive Value of Acute Neuroplastic Response to rTMS in Treatment Outcome in Depression: A Concurrent TMS-fMRI Trial. Am. J. Psychiatry. 179 (7), 500–508. 10.1176/appi.ajp.21050541 (2022). 35582784

[15] Granziera C. Quantitative magnetic resonance imaging towards clinical application in multiple sclerosis. Brain 144 (5), 1296–1311. 10.1093/brain/awab029 (2021). 33970206 PMC8219362

[16] Jackson J. B., Scrivener C. L., Correia M. M., Mada M. & Woolgar A. Conducting interslice stimulation for concurrent TMS-fMRI. J. Neurosci. Methods. 422, 110513. 10.1016/j.jneumeth.2025.110513 (2025). 40484287

[17] Joutsa J., Corp D. T. & Fox M. D. Lesion network mapping for symptom localization: Recent developments and future directions. Curr. Opin. Neurol. 35 (4), 453–459. 10.1097/WCO.0000000000001085 (2022). 35788098 PMC9724189

[18] Kaster T. S. Differential symptom cluster responses to repetitive transcranial magnetic stimulation treatment in depression. eClinicalMedicine, 55. (2023). 10.1016/j.eclinm.2022.101765 PMC9722479 36483268

[19] Kong G., Wei L., Wang J., Zhu C. & Tang Y. The therapeutic potential of personalized connectivity-guided transcranial magnetic stimulation target over group-average target for depression. Brain Stimulation: Basic. Translational Clin. Res. Neuromodulation. 15 (5), 1063–1064. 10.1016/j.brs.2022.07.054 (2022). 35931377

[20] Luber B. M. Using neuroimaging to individualize TMS treatment for depression: Toward a new paradigm for imaging-guided intervention. NeuroImage 148, 1–7. 10.1016/j.neuroimage.2016.12.083 (2017). 28062252 PMC5344760

[21] Lueckel M. Functional connectivity- and E-field-optimized TMS targeting: Method development and concurrent TMS-fMRI validation. Brain Stimulation: Basic. Translational Clin. Res. Neuromodulation. 16 (1), 189. 10.1016/j.brs.2023.01.223 (2023).

[22] Morriss R. Connectivity-guided intermittent theta burst versus repetitive transcranial magnetic stimulation for treatment-resistant depression: A randomized controlled trial. Nat. Med. 30 (2), 403–413. 10.1038/s41591-023-02764-z (2024). 38228914 PMC10878976

[23] Nagaraja N. Diffusion weighted imaging in acute ischemic stroke: A review of its interpretation pitfalls and advanced diffusion imaging application. J. Neurol. Sci. 425, 117435. 10.1016/j.jns.2021.117435 (2021). 33836457

[24] De Navarro L. I. High-sensitivity TMS/fMRI of the Human Motor Cortex Using a Dedicated Multichannel MR Coil. NeuroImage 150, 262–269. 10.1016/j.neuroimage.2017.02.062 (2017). 28254457

[25] Navarro de Lara L. I. New ultra-thin multichannel receive coil for concurrent TMS/fMRI experiments. Brain Stimul. 8 (2), 426–427. 10.1016/j.brs.2015.01.358 (2015).

[26] Oathes D. J. Combining transcranial magnetic stimulation with functional magnetic resonance imaging for probing and modulating neural circuits relevant to affective disorders. Wires Cogn. Sci. 12 (4), e1553. 10.1002/wcs.1553 (2021). PMC8521438 33470055

[27] Oathes D. J. Non-invasively targeting, probing and modulating a deep brain circuit for depression alleviation. Nat. Mental Health. 1 (12), 1033–1042. 10.1038/s44220-023-00165-2 (2023). PMC12919846 41726164

[28] Oathes D. J. Resting fMRI-guided TMS results in subcortical and brain network modulation indexed by interleaved TMS/fMRI. Exp. Brain Res. 239 (4), 1165–1178. 10.1007/s00221-021-06036-5 (2021). 33560448 PMC8521442

[29] Parmigiani S. Real-time optimization to enhance noninvasive cortical excitability assessment in the human dorsolateral prefrontal cortex. Clin. Neurophysiol. 174, 225–234. 10.1016/j.clinph.2025.02.261 (2025). 40148152

[30] Rafiei F., Safrin M., Wokke M. E., Lau H. & Rahnev D. Transcranial magnetic stimulation alters multivoxel patterns in the absence of overall activity changes. Hum. Brain. Mapp. 42 (12), 3804–3820. 10.1002/hbm.25466 (2021). 33991165 PMC8288086

[31] Riva-Posse P. A connectomic approach for subcallosal cingulate deep brain stimulation surgery: Prospective targeting in treatment-resistant depression. Mol. Psychiatry. 23 (4), 843–849. 10.1038/mp.2017.59 (2018). 28397839 PMC5636645

[32] Roalf D. R., Figee M. & Oathes D. J. Elevating the field for applying neuroimaging to individual patients in psychiatry. Translational Psychiatry. 14, 87. 10.1038/s41398-024-02781-7 (2024). 38341414 PMC10858949

[33] van Rooij S. Targeting the Amygdala in a TMS Clinical Trial for PTSD. Biol. Psychiatry. 95 (10), S31–S32. 10.1016/j.biopsych.2024.02.082 (2024).

[34] Seguin C. Communication dynamics in the human connectome shape the cortex-wide propagation of direct electrical stimulation. Neuron 111 (9), 1391–1401e5. 10.1016/j.neuron.2023.01.027 (2023). 36889313

[35] Soleimani G. Targeting VMPFC-amygdala circuit with TMS in substance use disorder: A mechanistic framework. Addict. Biol. 30 (1), e70011. 10.1111/adb.70011 (2025). 39783881 PMC11714170

[36] Sydnor V. J. Cortical-subcortical structural connections support transcranial magnetic stimulation engagement of the amygdala. Sci. Adv. 8 (25), eabn5803. 10.1126/sciadv.abn5803 (2022). 35731882 PMC9217085

[37] Tik M. Interslice TMS/fMRI enables continuous EPI during clinical rTMS and iTBS protocols. Brain Stimulation: Basic. Translational Clin. Res. Neuromodulation. 14 (6), 1723–1724. 10.1016/j.brs.2021.10.446 (2021).

[38] Tik M. Mapping TMS local and remote immediate effects by concurrent TMS/fMRI using a dedicated high-sensitivity coil array. Brain Stimulation: Basic. Translational Clin. Res. Neuromodulation. 10 (2), 489–491. 10.1016/j.brs.2017.01.432 (2017).

[39] Trapp N. T. Dorsolateral prefrontal cortex TMS evokes responses in the subgenual anterior cingulate cortex: Evidence from human intracranial EEG. bioRxiv: The Preprint Server for Biology, 2024.12.20.629857. (2024). 10.1101/2024.12.20.629857

[40] Woolgar A. Consensus guidelines for the use of concurrent TMS-fMRI in cognitive and clinical neuroscience. Nat. Protoc. 1–17. 10.1038/s41596-025-01182-4 (2025). 40555803

[41] Xia A. W. L. Instantaneous effects of prefrontal transcranial magnetic stimulation on brain oxygenation: A systematic review. NeuroImage 293, 120618. 10.1016/j.neuroimage.2024.120618 (2024). 38636640

